# Families’ Division of Labor and Social Networks in the 21st Century: Revisiting Elizabeth Bott’s Classic Hypotheses

**DOI:** 10.1177/0192513X18783230

**Published:** 2018-06-25

**Authors:** Jesper Rözer, Gerald Mollenhorst, Beate Volker

**Affiliations:** 1 University of Amsterdam, Amsterdam, Netherlands; 2 Utrecht University, Utrecht, Netherlands

**Keywords:** family, division of labor, personal network, marriage, Elizabeth Bott, PAIRFAM

## Abstract

In 1957, Elizabeth Bott argued that the organization of family and social networks are intertwined and that the structure and composition of social networks are associated with the ways in which spouses divide household and paid labor. While this idea became a classic in the literature addressing the division of labor, societies have changed tremendously in the past 50 years, and it has become far more common for spouses to divide their labor more equally. In addition, the causal direction is not clear: Do networks affect the division of labor or vice versa? We inquired as to the causal relationship using a large-scale longitudinal data set, collected in 2009/2010 and 2011/2012 (*n* = 2477; PAIRFAM [Panel Analysis of Intimate Relationships and Family Dynamics]). We found moderate support for the hypothesis that personal networks influence the division of labor in households, but there were stronger effects for the reverse—that is, that the division of labor affects network patterns, particularly for women.

In her now classic 1957 study titled *Family and Social Network*, Elizabeth Bott argued that social networks affect how people organize their families. She observed that spouses who both have a close-knit network outside their family had a clear division of tasks, while spouses who both had a less dense social network more equally shared tasks such as working, cleaning, and child rearing. Bott elaborated on this relationship between the organization of the family and the composition and structure of social networks, and she developed several hypotheses, of which the above mentioned is the most well-known: A close-knit, dense network results in segregated conjugal roles and a clear division of labor between husband and wife.

Although Bott’s hypotheses have become a classic example of how the division of labor between spouses and their social networks are intertwined (e.g., Bidart & Lavenu, 2005; Fuhse, 2009; Kalmijn, 2003; Kilduff & Brass, 2010; Plickert, Côté, & Wellman 2007; Wellman & Frank, 2001), the picture of families in her study seems a bit old-fashioned. With increased emancipation of women, the differences between men and women in terms of their division of labor and gender roles have declined (e.g., Altintas & Sullivan, 2016; Cotter, Hermsen, & Vanneman, 2011). At the same time, with increased individualization, people have become liberated from the constraints of their network, and they have more opportunities to pursue their own lifestyles—including their division of labor (e.g., Giddens, 1991). These developments raise the question of whether Bott’s arguments still hold.

In this study, we inquire into Bott’s hypotheses and empirically test them with new data. We improve on previous studies that tested Bott’s hypotheses (e.g., Blood, 1969; Giudici & Widmer, 2015; Ishii-Kuntz & Maryanski, 2003; Kapferer, 1973; Maryanski & Ishii-Kuntz, 1991; Treas, 2011; Turner, 1967; Udry & Hall, 1965; Wellman & Wellman, 1992) by being the first to put many of them to a rigorous test with large-scale panel data (the German PAIRFAM [Panel Analysis of Intimate Relationships and Family Dynamics] data) and by taking issues of causality into account by using a cross-lagged model. In short, we examine how families (i.e., the nuclear family of husband, wife, and their children) and the structure and composition of social networks (i.e., density, overlap, and composition) are intertwined in the 21st century.

## Family and Social Networks

According to Bott (1957), the social norms that existed in the 1950s implied that the family constituted the “backbone” of society. In spite of this, there were very few empirical studies on the ways in which individuals within families were embedded in larger social structures, such as in social networks and their residential neighborhood. Therefore, Bott decided to study 20 London families and explore the extent to which the organization of the home was associated with larger patterns in society. There appeared to be “a considerable variation in the role relationships between husband and wife” (Bott, 1957, p. 3). Families differed along the lines of a “segregated conjugal role relationship” and a “joint conjugal role relationship.” In a segregated conjugal role relationship, “husband and wife have a clear differentiation of tasks and a considerable number of separate interests and activities. They have a clearly defined division of labor into male tasks and female tasks” (Bott, 1957, p. 53). In a joint conjugal role relationship,

Husband and wife expect to carry out many activities together with a minimum of task differentiation and separate interests. They not only plan the affairs of the family together but also exchange many household tasks and spend much of their leisure time together. (Bott, 1957, pp. 53-54)

Initially, Bott tried to explain the conjugal role relationships with general sociological categories, such as social class, income, and occupational status. Although no clear statistical pattern has been observed among these characteristics, Bott refused to interpret the role relationships in terms of personality factors, thereby avoiding the reduction of group processes to psychological processes. Instead, she argued that sociological theories should explain the division of household tasks and paid labor between spouses (Bott, 1957), while focusing on the direct environment of the families—that is, their social networks. In particular, she addressed three network characteristics that should affect the division of paid labor and household tasks among spouses: network density, network composition, and network overlap. We discuss these three characteristics successively.

First, Bott (1957) observed a clear association between the conjugal role relationships and the density of the social network of the families:

Those families that had a high degree of segregation in the role relationship of husband and wife had a close-knit network . . . [while] families that had a relatively joint role-relationship between husband and wife had a loose-knit network. (p. 59)

The more close-knit or dense social networks are, the more friends, neighbors, and relatives will know one another well.

Bott argued that the degree of connectedness within personal networks affects how spouses shape the division of household and paid labor. If each of the spouses had a close-knit (but not overlapping) network, spouses would share tasks less equally, because informal social pressure is maximized in close-knit networks and people also help one another in cases of need (Bott, 1957). Hence, close-knit networks can provide substantial emotional satisfaction and support that may make individuals less dependent on their spouse for physical and emotional support as well as for help with household tasks, child rearing, and paid work. Consequently, those with close-knit networks live their lives relatively independent of their spouses and specialize either in household tasks and child rearing or in paid work, without needing much help from their spouse. In addition, in close-knit networks, norms with regard to task specialization spread more easily, and living up to norms is controlled (e.g., Bott, 1957, Chapter 7). In contrast, in joint conjugal role relationships, sparse-knit networks were prevalent. As a result, spouses had to get along and support each other, as there were little external sources of support. This argument constitutes Hypothesis 1: *A close-knit network leads to a segregated conjugal role relationship*.

Second, with regard to the other two network characteristics—that is, network composition and overlap—Bott observed that spouses with a segregated division of labor had nonoverlapping networks and argued that this was because men and women lived in their own social circles. For example, women were friends with their female neighbors; met them in the street, shops, and school yard; and occasionally observed them in their homes (Bott, 1957). In addition, female relatives often helped with the household and care of the children. The help of the mother and mother-in-law was particularly important as they had the important task of providing household advice and support (Bott, 1957).

Men in a segregated conjugal role relationship associated primarily with “friends” and colleagues they met through their work. They also formed contacts with male neighbors and relatives, but through their wives. They still mainly had contact with other men because other men had similar interests and entered similar places. For example, it was likely that men played soccer together or went to the pub after work. Spouses were not expected to become friends with the friends of their spouses because their interests differed too much. Instead, women became friends with women and men with men.

Couples with a less segregated division of labor were also assumed to be more integrated in their social contacts; because husband and wife were both active in many social contexts, such as the neighborhood and at work, they both drew their social contacts from a mixture of contexts and correspondingly formed relationships with neighbors, colleagues, relatives, and “friends” (Bott, 1957). Through their shared interests and activities, spouses developed mutual friends. For instance, at parties, women and men did not cluster together at opposite ends of the room but mixed and talked with everyone (Bott, 1957).

Thus, based on Bott’s study, we expect that *in families with a clear division of labor, women primarily associate with relatives and neighbors and less with colleagues* (Hypothesis 2a), *while men primarily associate with friends and colleagues and via their wives also have more contact with relatives and neighbors compared to men with a joint relationship* (Hypothesis 2b). Furthermore, we expect that *spouses who have a clear division of labor have more same-gender contacts* (Hypothesis 3) and *share fewer contacts with each other* (Hypothesis 4) compared with spouses who more equally share their household and paid labor responsibilities.

In contrast to the first hypothesis, Bott assumed no clear causal directions for these other hypotheses. However, she tends to say that the social network affects the division of labor among spouses (see Ishii-Kuntz & Maryanski, 2003; Wellman & Wellman, 1992), rather than the other way around. However, the implied causality between network density and the degree to which a conjugal relationship is segregated, as formulated in the first hypothesis, can be questioned. If people with a segregated role relationship are more likely to draw social contacts predominantly from one social context (e.g., men making friends at work), this increases the likelihood that their social contacts know one another and their networks become dense (Feld, 1981). This would mean that the structure and composition of networks are consequences of the way in which spouses divide paid labor and household tasks, instead of vice versa.

Bott (1957) assumed that her hypotheses were of “general relevance” (p. 9). Many of these hypotheses have become widely accepted, such as dense networks being more supportive and associated with a segregation of conjugal roles and women’s social networks containing more kin than those of men (e.g., Bidart & Lavenu, 2005; Fuhse, 2009; Kalmijn, 2003; Kilduff & Brass, 2010; Plickert et al., 2007; Wellman & Frank, 2001). Nevertheless, Bott’s picture of British families seems a bit old-fashioned. With the increased emancipation of women, gender roles have become more equal and differences between men and women in terms of education, labor market participation, and the division of household tasks have declined (e.g., Altintas & Sullivan, 2016; Cotter et at., 2011). As a consequence, the experiences of men and women may have become more equal and social contexts, such as the neighborhood or the workplace, have become less gender segregated. In addition, individuals and their networks are increasingly liberated from local constraints, geographical boundaries, and the claims of formerly ascribed social contacts, such as neighbors and (extended) family (Wellman & Frank, 2001). People are required to shape their own lives, maneuver through social space, and find social contacts that fit their lifestyle, instead of adopting a lifestyle—including division of labor—in line with the demands of institutions such as the state and church and their social network (e.g., Giddens, 1991). A potential consequence of these developments is that social networks currently have a smaller influence on how spouses divide their tasks and that the importance of norms and gender-specific social contexts has decreased. This raises the question of whether Bott’s arguments still hold in the 21st century. It is plausible that the network is a consequence of the division of labor rather than its cause. This might hold particularly because norms of gender-divided tasks at home have eroded and networks in general are alleged to be less dense in modern times (cf. Fischer, 1982). In the following, we put Bott’s ideas and our arguments to a test.

## Data and Operationalization

### The PAIRFAM Data

We use information from the German Family Panel Data (PAIRFAM; Huinink et al., 2011; Nauck, Brüderl, Huinink, & Walper, 2013). This data set is particularly suitable for our research because it is one of the few large-scale surveys that include an extensive social network questionnaire as well as questions about conjugal role relationships. From the six waves that are available thus far, we use the second and fourth waves because they were the only two that included a largely comparable network module.

The first wave of the PAIRFAM was carried out between September 2008 and May 2009, the second between October 2009 and May 2010, the third between October 2010 and April 2011, and the fourth between October 2011 and May 2012. Respondents were born in 1971 to 1973, 1981 to 1983, or 1991 to 1993, such that they were between 16 and 39 years old in Wave 2 and between 18 and 41 years old in Wave 4. This age range corresponds with Bott’s age range of “normal” London families with children below 10 years of age.

For the first wave of the PAIRFAM, 42,000 addresses were randomly drawn from the population registers of 343 randomly selected municipalities in Germany. With a response rate of 36.9%, the first wave included 12,402 respondents.^1^ Fortunately, the nonresponse bias was limited: frequency distributions of gender, provinces, city size, and marital status were similar to the Mikrozensus 2007, which was a compulsory survey for a 1% sample of the German population (Huinink et al., 2011). The second wave included information about 9,069 respondents (i.e., 73.1% of the first wave). From these respondents, 6,640 participated in the fourth wave (i.e., 70.4% of the second wave). These respondents differed slightly from the respondents in the first wave on characteristics such as income and education. We control for these variables in the analyses. To examine the influence of conjugal roles, only respondents who lived together with a romantic partner in both waves are selected (*n* = 2477, 37.3%).

### Measurements

#### Conjugal Roles

To measure conjugal roles, respondents were asked about their division of household labor and the number of hours they worked. Specifically, they were asked,

To what extent do you and [Name of Partner] share duties in the following domains? If you have a housemaid, nanny, or similar household help, then refer in your answers only to the portion of work done by you and/or your partner.

The domains were caring for the children, housework, shopping, home and car repairs, and finance. Possible response categories were (1) “(almost) completely my partner,” (2) “for the most part my partner,” (3) “split approximately 50/50,” (4) “for the most part me,” and (5) “(almost) completely me.” We rescaled the answering categories ranging from 0 = *joint* (split approximately 50/50) to 2 = *segregated* (almost) completely my partner/I). If there was segregation on one of the five duties, it was almost always the wife spending more time on childcare, housework, and shopping and the husband spending more time on the car and finance. Furthermore, the number of hours the respondent and the spouse worked was investigated. Response categories were rescaled to 0 = *spouses worked approximately equal hours* (less than 10 hours difference), 1 = *one worked between 10 and 30 hours less/more than her or his spouse*, and 2 = *one worked many hours more/less than her or his spouse* (more than 30 hours difference). Hence, another scale was created ranging from 0 = *joint* to 2 = *segregated*. A segregation in work hours almost always implied that the husband worked full time and the wife had no or only a marginal job. To construct the final scale, we weighted each item with the average amount of time German inhabitants spend on these paid work and household tasks, using the German Time Surveys (Zeitverwendungserhebung). In 2012/2013, Germans between the ages of 30 and 44 years (relevant information was lacking for younger respondents) on average spent 4.25 hours on paid work, 2.22 hours on housework, 1.47 hours on childcare if they had children, 0.5 hours on shopping, and 0.23 on repairs per day. Because no specific information was available about finance, we excluded this item. We could not split the time spent on housework for people with and without children, although people with children will probably spend more time on it. For couples with and without children, we calculated what percentage of time they spent on each task. Accordingly, for people without children the scale is constructed as follows: .59*paid work + .30*housework + .07*shopping + .03*repairs. For people with children the scale is constructed as follows: .47*paid work + .19*child care + .25*housework +.06*shopping + .03*repairs. Descriptive statistics are presented in Table 1.

**Table 1.** Descriptive Statistics.

			Women					Men				
				Wave 2		Wave 4			Wave 2		Wave 4	
	Min	Max	*r* W2/W4	*M*	*SD*	*M*	*SD*	*r* W2/W4	*M*	*SD*	*M*	*SD*
*Conjugal roles*												
Degree of segregation	0	2	.53	1.01	0.59	1.02	0.58	.54	0.97	0.56	0.95	0.57
Joint (score 0-00.66)	0	1	.45	0.31	0.46	0.30	0.46	.43	0.33	0.47	0.34	0.47
Mixed (score 0.67-1.33)	0	1	.18	0.34	0.47	0.35	0.48	.29	0.34	0.47	0.34	0.48
Segreg. (score 1.34-2)	0	1	.37	0.35	0.48	0.35	0.48	.42	0.33	0.47	0.32	0.47
*Social network*												
Density	−3.09	1.83	.57	0.09	0.58	0.04	0.57	.52	0.04	0.66	0.01	0.63
Number of kin	0	11	.42	0.78	1.04	0.89	1.24	.39	0.74	1.1	0.71	1.16
Number of neighbors	0	7	.42	0.29	0.73	0.36	0.77	.30	0.24	0.66	0.30	0.76
Number of friends	0	8	.54	1.8	1.5	2.01	1.61	.48	1.3	1.42	1.48	1.52
Number of colleagues	0	8	.46	0.33	0.72	0.41	0.84	.40	0.33	0.76	0.46	0.96
Percentage of same gender	0	1	.33	0.85	0.22	0.85	0.24	.32	0.78	0.26	0.80	0.29
Average closeness of the spouse to social contacts	0	4	.44	1.97	0.93	0.98	0.79	.39	2.08	0.90	1.02	0.80
*Control variables*												
Having a child/children aged												
0-1 years	0	1	.03	0.19	0.39	0.16	0.36	.04	0.21	0.41	0.18	0.38
2-4 years	0	1	.36	0.31	0.46	0.30	0.46	.44	0.29	0.45	0.33	0.47
5-10 years	0	1	.67	0.43	0.50	0.47	0.50	.70	0.37	0.48	0.43	0.50
11-17 years	0	1	.71	0.22	0.42	0.31	0.46	.74	0.16	0.37	0.24	0.42
18+	0	1	.68	0.04	0.20	0.08	0.27	.59	0.02	0.13	0.04	0.19
Age	16	41	1.00	33.31	5.12	35.29	5.12	1.00	33.83	4.93	35.79	4.93
Migration status												
Native	0	1	1	0.71	0.43	0.71	0.43	1	0.79	0.41	0.79	0.41
First generation	0	1	1	0.15	0.35	0.15	0.35	1	0.12	0.32	0.12	0.32
Second generation	0	1	1	0.08	0.27	0.08	0.27	1	0.09	0.28	0.09	0.28
Income	0	6	.57	2.91	1.49	3.19	1.35	.5	3.03	1.33	3.47	2.61
Education	0	7	.99	4.14	1.63	4.16	1.65	.98	4.35	1.68	4.35	1.68
Distance to parents	0	5	.83	1.78	0.93	1.85	0.99	.85	1.68	0.94	1.71	0.98
Mother alive	0	1	.94	0.91	0.29	0.90	0.30	.92	0.91	0.29	0.89	0.31
Father alive	0	1	.92	0.81	0.39	0.78	0.41	.94	0.80	0.40	0.78	0.41

*Note.* 1,377 women and 940 men who participated in Wave 2 and Wave 4 lived together with a partner. *r* = correlation between the waves.

*Source.* PAIRFAM Waves 2 and 4.

#### The Social Network

To measure the social networks, we use answers to the following three “name-generating questions”: “With whom do you share personal thoughts and feelings or discuss things that you will not discuss with anyone else?” “Which persons do you regularly meet for activities (e.g., sports), or when you go out (cinema, dancing), or when you just want to talk with someone?” “Who helps you whenever you need information or concrete advice on practical matters?”^2^

After respondents mentioned their social contacts, name-interpreting questions were asked. First, respondents were asked about their relationship to the social contact, to allow us to construct the composition of their personal network in terms of the number of *relatives* (i.e., mother, father, stepparent, or current partner of father/mother, mother’s or father’s ex-partner, partner’s mother/father, common child with partner, own/biological child, sibling, grandparent, aunt/uncle, cousin, nephew, niece, partner’s sibling, partner’s grandparent, another relative), the number of *neighbors* (i.e., nonrelatives who are met in the neighborhood), and the number of *colleagues* (i.e., nonrelatives who are met through work) included in their social network. Respondents were forced to choose how they were related to each specific network member—that is, one personal contact could not be a neighbor and relative at the same time. Because no specific questions were asked about whether people regarded nonrelatives as friends, we consider them *friends* if respondents felt close to them (scoring at least 4 on a 5-point scale). In other words, the category “friends” is a subset of all close nonrelatives, irrespective of whether they are neighbors or colleagues.

We use three proxies for network density: closeness to the respondent, the duration of the contact (in years), and the number of different “contexts” in which personal contacts are met.^3^ Regarding the latter, for each relationship, respondents could choose one of the following contexts: family, work, the neighborhood, through one’s (ex-)partner, education, a club, a leisure activity, the Internet, and other contexts. Respondents could only indicate that they met contacts in one context. We assume that network density increases with the strength of relationships, with the duration of relationships, and with smaller numbers of social contexts in which the total set of the respondent’s contacts are met. This is a result of all three of these characteristics increasing the likelihood of simultaneous meetings with multiple network members, leading to greater network closure (Feld, 1981; Granovetter, 1973). We use the natural logarithm of the number of years that respondents knew their social contacts because the likelihood that an individual’s social contacts became friends if they had not already become so decreases over time. To construct the final measure for network density, we calculated the average of the *z* scores of the three items on closeness, duration, and number of contexts.

We use the gender of respondents’ social contacts to construct the *proportion of same-gender contacts in the network*. Furthermore, we included the *average closeness of the spouse to the social contact*. Closeness of the spouse to the social contact is measured on a 5-point scale, ranging from 0 to 4.

#### Control Variables

In the multivariate analyses, we control for the respondent’s *age*, and *whether or not the respondent had a child/children in several age categories* as standard demographic variables. We include the following age categories: 0 through 1 (a baby), 2 through 4 (preschool), 5 through 10 (primary education), 11 through 17 (secondary education), and 18+ (adult).^4^ Furthermore, we control for *migration status* (native, first generation, second generation) as migrants typically have a more segregated division of labor and a different network structure than natives (e.g., more kin) (Lancee, 2011). We control for *education* because more highly educated people might have different networks and more progressive norms regarding their division of labor than their less educated counterparts (McPherson, Smith-Lovin, & Brashears, 2008). We also control for *income* because income can be used to outsource household tasks and child care. Furthermore, we control for whether the *father and mother are still alive* and for the *distance that people live from their parents*, because distance may decrease the likelihood that parents (and family that live close to the parents) help respondents. Consequently, people depend more on other network members or their spouse for support in the latter case, leading to a more joint conjugal role relationship (Bott, 1957).

### Analytical Strategy

We tested our hypotheses in three steps. First, we relied on descriptive statistics to describe the social network of respondents with a joint, mixed, and segregated conjugal role relationship. This method—which corresponds to the one Bott used to support her (causal) claims—provided deeper insight into how networks of people with a joint, mixed, and segregated division of labor differ in absolute numbers.

Second, we used ordinary least squares (OLS) regression models to show how conjugal roles and the social network are associated, controlling for important characteristics that might bias bivariate relationships, such as the number of children people have. They provided the most intuitive information about how much variance can be explained by each variable. We used the fourth wave for these cross-sectional descriptions and OLS regression models because it is the most recent.

Third, we used longitudinal cross-lagged models while employing the second (T1) and fourth (T2) waves to partially account for the causality between conjugal roles and the social network. In particular, we estimated cross-lagged models in which conjugal role relationships at T2 are predicted by the network variables at T1, controlled for the conjugal role relationship at T1. Likewise, we estimated cross-lagged models in which the network variables at T2 are predicted by the conjugal role relationship at T1, controlled for the network variables at T1. The reasoning behind this procedure is that independent variables measured at an earlier time point (X_T1_) predicted an outcome at a later time point (Y_T2_), controlled for the earlier value of the outcome variable (Y_T1_) such that the association between the independent variables (X_T1_) and the outcome variable (Y_T2_) is likely causal in nature. Linear variables (i.e., division of labor, network density, support from parents, number of times partner is mentioned in name-generating questions, average closeness of partner to social contacts, and percentage of same-gender social contacts) are estimated with OLS regression, while count variables (i.e., number of family members, neighbors, colleagues, and friends) are estimated with negative binominal regression models.

We use multiple imputation for handling missing data, particularly missing values on the variables “average closeness of the partner to one’s social contacts” (14.9% of all cases in Wave 2 and 12.5% in Wave 4), “percentage of network members with the same gender” (9.6% of all cases in Wave 2 and 7.1% in Wave 4), and “income” (8.7% of all cases in Wave 2 and 6.7% in Wave 4). The first two variables have so many missing values because they could only be computed for respondents with at least one social contact who was not a partner or child. Other variables had less than 5% missing values. To impute the missing values, we created five imputed data sets and predicted missing values for all variables in our model, including if possible the respondent’s answer to the same question in the previous/next wave. The results appeared to be robust for not imputing the missing values; listwise deletions led to similar results as those when the missing values were imputed.

## Results

We start with further describing the dependent and independent variables (see also Table 1). We divided our measure of the degree of a segregated role relationship into three equal parts: Respondents who scored between 0 and 0.66 were considered to have a joint role relationship, respondents who scored between 0.67 and 1.33 were considered to have a mixed role relationship, and those who scored between 1.34 and 2 were considered to have a segregated role relationship. In 2011/2012 (wave 4), around a third of the respondents had a mixed role relationship (35% of women, 34% of men), around a third had a joint role relationship (30% of women, 34% of men), and around a third had a segregated role relationship (35% of women, 32% of men).

Table 2 shows that the density of the network was highest for women with a mixed and segregated role relationship and highest for men with a joint or mixed role relationship. There was little difference among women with a joint, mixed, or segregated role relationship in the number of kin they had in their network. The number of close kin relationships was larger for men in a joint and mixed role relationship than for men in segregated role relationship. For men and women, the number of neighbors with whom they were close with was relatively small and smallest for those in joint relationships. Women with a mixed role relationship had slightly more friends than women with a segregated or joint role relationship, while men had more friends in a joint than in a mixed or segregated role relationship. The number of close relationships with colleagues was relatively high if men and women had a joint role relationship. Social networks were clearly gender segregated (figures are larger than .5). Women with a joint role relationship had more opposite-gender personal contacts than women with a mixed or segregated role relationship, while men with a joint or mixed role relationship had more opposite-gender contacts than men with a segregated role relationship. Finally, the average closeness of an individual’s spouse to other close contacts was slightly higher for women with a mixed role relationship. The spouses of men who had a mixed or segregated role relationship were somewhat closer to the men’s social contacts than the spouses of men who had a joint role relationship.

**Table 2.** Average Network Measures per Joint, Mixed, and Segregated Conjugal Role Relationships.

	Women				Men			
	Joint	Mixed	Segregated	Total	Joint	Mixed	Segregated	Total
Density	−0.07	0.08	0.08	0.04	−0.02	−0.04	−0.10	−0.05
Number of kin	0.85	0.91	0.89	0.89	0.77	0.73	0.64	0.71
Number of neighbors	0.24	0.38	0.45	0.36	0.24	0.33	0.33	0.30
Number of friends	2.00	2.06	1.99	2.02	1.61	1.43	1.39	1.48
Number of colleagues	0.56	0.38	0.30	0.41	0.50	0.40	0.42	0.44
Percentage same-gender contacts	0.81	0.87	0.86	0.85	0.80	0.78	0.84	0.80
Average closeness of spouse to social contacts	1.81	1.85	1.79	1.82	1.87	1.92	1.82	1.87

*Source.* PAIRFAM Wave 4.

Table 3 also presents the association between the family and the social network but controls for several conditions and treats conjugal relationship as a continuous variable running from 0 = *joint* to 2 = *segregated*. Having one or more children in various age-groups in the household is a particularly strong predictor for having a segregated role relationship. The effect of a child is particularly strong when it is still a baby and becomes weaker afterward. For women having a grown-up child (age 18+) is even negatively associated with a segregated role relationship. As presented in Model 2, if we remove this variable from the full model (see M3), the explained variance decreases by 10.1% for women and 12.6% for men. By comparison, all other variables together only explain 6.2% and 4% for women and men, respectively (see M2). Age and being a first-generation migrant are also positively associated with a segregated role relationship. For women, these variables, however, have little explanatory value (1.2%). By contrast, for men, leaving these variables out reduces the explained variance by another 3%. The network variables together explain approximately 1% of the variation for men and 2.4% for women.

**Table 3.** OLS Regression Predicting Degree of Segregated Conjugal Relationship.

	Women			Men		
	M1	M2	M3	M1	M2	M3
*Intercept*	.363*	.625**	.393*	.393*	.273	.219
*Control variables*						
Having a child/children aged						
0-1 years	.433**		.407**	.426**		.428**
2-4 years	.201**		.196**	.208**		.203**
5-11 years	.203**		.185**	.199**		.194**
12-17 years	.111**		.093*	.108*		.104*
18+	−.131*		−.126*	.030		.024
Age	.014**	.012**	.014**	.008^∼^	.015**	.009^∼^
Migration status (ref = native)						
First generation	.158**	.136**	.145*	.235**	.259**	.230**
Second generation	.010	.026	.013	.070	.077	.070
Income	−.024	−.020	−.023	−.006	−.005	−.005
Education	−.010	−.011	−.010	.015	.016	.018
Distance to parents	−.025	−.007	−.017	−.017	−.029	−.022
Mother alive	.022	−.008	.010	−.029	−.010	−.013
Father alive	.021	.012	.010	−.007	−.012	−.003
*Social network*						
Density		.132**	.121**		−.028	−.029
Number of kin		−.003	−.007		−.002	.009
Number of neighbors		.091**	.075**		.055*	.058*
Number of friends		−.002	.003		−.015	−.008
Number of colleagues		−.077**	−.050**		−.028	−.023
Percentage same-gender contacts		.205*	.116^∼^		.141^∼^	.110
Average closeness of spouse to social contacts		−.045*	−.049**		.020	.016
Adjusted *R*^2a^	.159	.062	.163	.140	.040	.166

a Mean 5 imputed models.

*Source.* PAIRFAM Wave 4.

∼ *p* < .10. *p < .05. **p < .01 (2-tailed).

Although they are small in magnitude, some (network) effects remain significant in the full model (see M3).^5^ For women, in line with Hypothesis 1, network density is positively associated with a segregated role relationship. Furthermore, in line with Hypothesis 2, having more contact with neighbors and less with colleagues is associated with a segregated division of labor. However, in contrast to Hypothesis 2a, contact with relatives is not significantly associated with a segregated division of labor. In line with Hypothesis 3, the percentage of same-gender contacts is (borderline) significantly associated with a segregated division of labor. And in line with Hypothesis 4, the average closeness of the partner to one’s social contacts is negatively associated with a segregated division of labor. For men, few effects are significant. There is only partial support for Hypothesis 2b: The number of close relationships with neighbors in their social network is positively associated with segregated roles.

Table 4 presents the cross-lagged models for women. The lagged effects of the family and network variables are presented below the line. Unsurprisingly, all lagged effects of the dependent variable are significant and explain most variance. For example, a 1-point higher score on the conjugal role relationship scale at T1 is associated with a .492 higher score at T2 (see column 1), and density at T1 is associated with a .390 higher score at T2 (see column 2).

**Table 4.** Cross-Lagged Model Predicting Degree of a Segregated Conjugal Relationship and Several Network Measures (for Women).

	Conjugal role	Density	Kin	Neighbor	Friends	Colleagues	Same gender	Spouse close
	1	2	3	4	5	6	7	8
Intercept	.324*	−.137	−.453	−2.128**	.139	−1.156*	.503**	1.142**
Having a child/children aged								
0-1 years	.015	−.075^∼^	−.156	0.048	−.031	−0.024	.011	−0.062
2-4 years	.104**	−.048	.002	0.062	.031	−0.054	−.003	−0.021
5-11 years	.037	−.039	−.115	0.175	−.049	−0.136	.003	−0.075
12-17 years	.020	.003	.082	0.386**	−.074	0.042	−.001	0.035
18+	−.083	.158*	.716**	−0.608	−.246^∼^	0.037	−.015	0.191
Age	.001	.003	−.017*	−−0.005	−.002	−0.001	−.001	0.004
Migration status (ref = native)								
First generation	.118**	.185**	.164	−0.166	−.050	−0.328	−.013	0.201**
Second generation	.006	.068	−.105	−0.107	.045	0.123	−.025	0.032
Income	.012	.007	−.003	0.029	.013	0.018	−.001	−0.006
Education	−.002	.006	.030	0.011	.065**	0.101**	.001	−0.040*
Distance to parents	−.027^∼^	−.048**	−.002	0.124*	−.013	0.097^∼^	−.002	−0.024
Mother alive	−.035	.037	.192	0.117	−.126*	−0.270	.011	−0.008
Father alive	.032	.008	.033	0.022	.015	−0.226^∼^	.033^∼^	0.004
Lagged dependent variable^a^	.492**	.390**	.313**	0.652**	.237**	0.545**	.347**	0.417**
Conjugal role								
Degree of segregation		.072*	.140*	0.273**	.062^∼^	−0.181^∼^	.036**	−0.026
*Social network*								
Density	.019							
Number of kin	.004							
Number of neighbors	.005							
Number of friends	.007							
Number of colleagues	−.036^∼^							
Percent same gender	.098							
Average closeness of the spouse to social contacts	.014							

*Note.* Dependent variable is measured in Wave 4 and independent variables in Wave 2. Models 3 through 5 are negative binominal regression models; the others are OLS regression models.

a Degree of having a segregated role relationship in Wave 2 (in M1), the number of kin in Wave 2 (in M2), and so on.

*Source.* PAIRFAM Waves 2 and 4.

∼ *p* < .10. *p < .05. **p < .01 (2-tailed).

In column 1, we test whether the network measures at T1 can explain the degree of a segregated role relationship at T2, while controlling for the degree of segregated roles at T1. We start with the relationship about which Bott clearly formulated a causal relationship—that is, that a close-knit network leads to a segregated division of labor. In contrast to this hypothesis, this relationship is not significant. Most other network variables are also not significant, indicating that the social network has little effect on how spouses divide their labor. The only effect that is (borderline) significant is that of the number of colleagues. Having a greater number of colleagues in the network at T1 is associated with a less segregated division of labor at T2.

In columns 2 through 8, we test for reverse causality. The initial degree to which a role relationship is segregated at T1 predicts several network measures at T2, controlled for the network measures at T1. A segregated role relationship at T1 predicts a more close-knit network at T2. Furthermore, women who had a more segregated role relationship at T1 reported more close relationships with relatives, neighbors, friends, and other women and fewer relationships with colleagues at T2. Additional analyses indicate that the decrease in contact with colleagues among women with a segregated role relationship was because these women had a paid job; and this effect becomes insignificant once we control for whether women were working and the number of hours they worked (models available on request).

Table 5 shows the results for men. In line with Table 3, there are little associations between the network measures and how spouses divided their labor. Column 1 shows that only having more contact with colleagues at T1 is (borderline significantly) associated with a more segregated division of labor at T2, controlled for the degree of role segregation at T1. Columns 2 through 8 show that how men divided household chores and paid labor with their spouse has no significant effect on the composition and density of their network.

**Table 5.** Cross-Lagged Model Predicting Degree of a Segregated Conjugal Relationship and Several Network Measures (for Men).

	Conjugal role	Density	Kin	Neighbors	Friends	Colleagues	Same gender	Spouse close
	1	2	3	4	5	6	7	8
Intercept	.213	−.028	.128	−1.829*	−.045	−.903	.681**	1.408**
Having a child/children aged								
0-1 years	.018	−.005	.107	−.507*	−.135^∼^	−.388*	.008	.036
2-4 years	.068^∼^	−.020	−.233^∼^	.076	−.032	−.177	.003	−.052
5-11 years	.060	.003	.124	.216	.028	.046	.007	.039
12-17 years	−.023	.070	.174	−.223	−.151	−.055	−.044	−.042
18+	.084	−.016	.166	.569	.221	.470	−.035	.128
Age	.002	−.001	−.044**	−.001	−.012^∼^	−.007	−.004^∼^	−.004
Migration status (ref = native)								
First generation	.170**	.046	−.207	.066	−.110	−.536*	.031	.090
Second generation	.085	.094	−.055	.223	.107	−.177	.052	−.057
Income	.006	−.007	−.040	−.089	.036	.051	−.002	.004
Education	.017	.004	.033	.040	.056**	.023	−.003	−.020
Distance to parents	−.014	−.056**	.003	.204*	.023	.117^∼^	.008	−.042
Mother alive	−.070	.060	.377	−.174	.178	.040	−.011	−.033
Father alive	−.015	.057	.222	−.116	−.042	−.345*	−.017	−.054
Lagged dependent variable^a^	.509**	.404**	.322**	.755**	.270**	.543**	.337**	.388**
*Conjugal role*								
Degree of segregation		−.019	−.056	.238	.014	−.042	.012	.036
*Social network*								
Density	.006							
Number of kin	.008							
Number of neighbors	−.029							
Number of friends	−.001							
Number of colleagues	.046^∼^							
Percent same gender	.100							
Average closeness of the spouse to social contacts	.007							

*Note.* Dependent variable is measured in Wave 4 and independent variables in Wave 2. Models 3 through 5 are negative binominal regression models; the others are OLS regression models.

a Degree of having a segregated role relationship in Wave 2 (in M1), the number of kin in Wave 2 (in M2), and so on.

*Source.* PAIRFAM Waves 2 and 4.

∼ *p* < .10. *p < .05. **p < .01 (2-tailed).

## Conclusion and Discussion

In the 1950s, Elizabeth Bott (1957) examined how personal networks and the structure of families are intertwined. She developed several hypotheses addressing the association between the division of household chores and paid labor among spouses, on the one hand, and the composition and structure of their personal networks, on the other. These hypotheses were meant to be of “general relevance” (Bott, 1957, p. 9). Although many have become widely accepted, no study tested these hypotheses fully and adequately by accounting for reversed causality. Indeed, with our data from 2009/2010 and from 2011/2012, we cannot test whether Bott’s hypotheses were supported at the time she wrote her book; however, we can determine whether the expected associations are observed currently and whether family influences the social network, or vice versa.

Using two waves of the German PAIRFAM data set, we found that only about a third of the respondents indicated that they almost equally shared tasks such as the household chores, raising the children, and earning income, while approximately two third indicated having “some” division or segregation of these tasks. Bott argued that having a close-knit personal network is an important reason why spouses divide their paid labor and household tasks. Such dense networks offer considerable support, which enables spouses to perform their tasks separately. In line with Bott and several recent studies that tested her hypothesis (e.g., Giudici & Widmer, 2015; Treas, 2011), our descriptive findings suggested that for women network density is associated with a more segregated division of labor. However, the cross-lagged models revealed that network density does not affect the division of labor for either women or men. In other words, we conclude that having a dense social network currently does not constrain the ways in which spouses divide paid labor and household tasks. Dense networks may currently be less likely to push conservative values, as public opinion has grown less traditional (Fahlén, 2016; Treas, 2011). For women, however, we found some indication for reverse causation; their networks tend to be more close-knit if they had a more segregated division of labor.

Bott further anticipated that the division of labor would be associated with the composition of personal networks. Our cross-lagged models did provide some support for that particularly for women. Women who were in a more segregated role relationship gained more close relationships with relatives, neighbors, friends, and other women. These findings may be partly due to meeting opportunities and shared interests. Moreover, women in a segregated role relationship may have more spare time than women in a joint relationship because the latter are in practice often still largely responsible for the household next to their paid job (Bianchi, Sayer, Milkie, & Robinson, 2012). Furthermore, we found that for women, a more segregated role relationship resulted in fewer close contact with colleagues. This was because these women worked (more hours) such that they had more opportunities to associate with colleagues. At the same time, we found that more close contacts with colleagues led to a less rigid division of labor for women. This suggests that colleagues encouraged women to more equally divide tasks with their spouse and hence probably to work more hours. Moreover, having more contact with colleagues may make women feel comfortable at work and spending more time and energy at work as compared with the household, resulting in a more equal division of tasks with their spouses. In contrast, when women have few close contacts at work, this may lead them to withdraw from work and to focus more on raising the children and the household chores.

Interestingly, for men, the social network and their division of labor were hardly intertwined. The only indication that the division of labor is affected by the network patterns for men was that more close contact with colleagues is positively associated with being in a segregated role relationship. How men divided their labor with their spouse had also no significant effect on their social life. This is in contrast to that of women. Apparently, the social lives of women are more regulated by how much time they spend on paid labor, household chores, repairs, and child care, than the social lives of men (Fahlén, 2016; Pollmann-Schult, 2017).

We can only speculate why this is exactly the case. It may be that women can less freely maneuver when they work, take care of their children, or do household chores than men. This would be in line with the finding that women more often prioritize children and the household above other things, while at the same time women often accept the idea that men’s careers are of primary importance (Chesley, 2017; Schrock & Schwalbe, 2009). A classic example of this is given by Hochschild and Magung (1989). They showed that fathers were less likely than mothers to pick up their children from school when their children are sick, even when both the fathers and the mothers were at work and had agreed to equally share tasks. Similar “bargaining” mechanisms between fathers and mothers may be at work in other situations. For instance, whether the father or the mother is most likely to call in the nanny (Cheung & Lui, 2017) or who is most likely to pick up a child from day care when both parents have to work (Tammelin, Malinen, Rönkä, & Verhoef, 2017). General expectations about parental roles of fathers and mothers may further strengthen these patterns (Giudici & Widmer, 2015).

Another explanation why only women adapt their social networks is that women seem to try more often to “fit in” at work, while men rather avoid to fit in “at home” because they often feel uncomfortable in a “women’s world” and want to keep up their masculinity (Connel & Messerschmidt, 2005; Schrock & Schwalbe, 2009). As a consequence, women, for example, often become friends with male colleagues and sometimes even keep back from female workers at work (the so-called queen bee effect; e.g., Derks, Van Laar, & Ellemers, 2016), while men often hesitate to become friends with the mothers at the school yard (Doucet, 2006).

But irrespective of its causes, the finding that women’s social lives are more regulated by the division of labor than that of men may have important consequences. For example, it can explain “second-shift” claims of overburden of women because it implies that the division of labor puts more constraints on women than on men—net of whether they equally divide their labor or not (e.g., Bianchi et al., 2012). Moreover, because women seem to adapt their social networks in line with the tasks they perform—often child rearing and the household—they may often have social networks that can offer little support in other domains such as the labor market (McDonald, Lin, & Ao, 2009; Morris, Teevan, & Panovich, 2010). Thus, the constraints a division of labor puts on women’s network may spill over into other domains and can contribute to existing gender inequality.

Some data limitations may have affected our findings. First, it has been shown that the degree to which spouses divide household and paid labor tasks is often underestimated and differs between men and women (e.g., Hochschild & Magung, 1989; Yavorsky, Kamp, Dush, & Schoppe-Sullivan, 2015; Young, Wallace, & Polachek, 2015). As a consequence, we may not have distinguished respondents who have an equal division of labor with their spouse from those who actually underestimate their inequalities in the division of labor. This may lead to an underestimation of the effects of the division of labor. Second, we combined several items that measured the amount of time men and women spend on paid work, household chores, and raising the children, with each of these factors possibly having separate effects on network characteristics. Third, we employed several proxies to measure network characteristics. For example, because network density was not measured straightforwardly in the questionnaire, we utilized three proxies to jointly measure this network characteristic. A more direct measurement may have more accurately tested the expected association between network density and the division of labor. Fourth, although we used cross-lagged models to get an idea of the causal relationships, some relationships may still be spurious and be explained by unmeasured confounders. For example, if those who are in a segregated role relationship generally move less frequently and thus stay longer in their residential neighborhood, this may (partly) explain their higher number of local contacts. Fifth, because Bott did not elaborate extensively on every expected association, or on the mechanisms that should drive these associations, we cannot be completely sure whether we have interpreted Bott’s work correctly in every sense. We endeavored to stay as close as possible to her text and ideas while directly quoting her and citing the pages from which we deduced her hypotheses. Related to this, as Bott reasoned herself, and is supported by Giudici and Widmer (2015), how spouses divide their labor may particularly affect social networks most when people get children.

We conclude that at the start of the 21st century, the division of paid labor and household tasks and the structure and composition of the social networks of spouses are (still) interrelated. Social networks do affect how couples divide their household tasks and paid labor in some respects: Women who have more close contacts with colleagues tend to divide their tasks more equally with their spouse, while men who have more close contacts with colleagues tend to divide their tasks less equally with their spouse. Accordingly, we agree with Bott that to understand how husbands and wives divide their paid labor and household tasks, we should not only look at well-known explanations, such as a need for efficiency based on the complementary role specialization (e.g., Becker, 1981), economic dependency and bargaining power (e.g., Gupta, 2007), or gender norms, cultural expectations, and “doing gender” (e.g., Berk, 1985; Davis & Greenstein, 2009), but also at the structure and composition of their social networks.

But we also conclude that the influence of social contacts on the division of labor is remarkably weaker than Bott observed in the 1950s. This is probably partly because she did not take into account reversed causality but maybe also because social networks currently are less likely to impose traditional gender norms. Instead, we find relatively more support for the argument that people currently shape their own lives, maneuver through social space, and find the social contacts that fit their lifestyle—including the division of paid labor and household tasks—which are in line with the characteristics of their social network. Interestingly, this is particularly the case for women, showing that how much they work and take care of the children and the household drives their social lives more than those of men. Accordingly, we conclude that to understand the composition and structure of personal networks, researchers should also look at how people divide their paid labor and household tasks with their spouses, because the tasks people perform create specific interests and needs, time and energy constraints, and (meeting) opportunities, which together affect whom they meet and with whom they have close interpersonal contact.

## Notes

1. Response rates below 40% are common in Germany, and the PAIRFAM is therefore not unusual in this respect.

2. Name-generating questions about quarrels or conflict were not used because they indicate sour social contacts. In total, up to 30 social contacts could be named. Approximately 40% of the variation in personal contacts could be attributed to interviewer effects. Exact binominal probability tests (Païk & Sanchagrin, 2013) showed that in Wave 2 and Wave 4 the results were probably contaminated in 4.1% and 3.6% of the cases, respectively. In total, we excluded 6.5% of our respondents, ending up with 2,317 respondents.

3. Spouses are not regarded as a social contact in any of the measures. Respondents were not asked about closeness with their parents and children, but we assume that people are very close to them. Respondents were not asked how long they know their family members, as it can be considered that they knew most of them all their life. The gender of children in the network was not investigated, but this happened only in 1.0% of the cases in Wave 2 and in 2.1% of the cases in Wave 4.

4. Additional analyses (not presented but available on request from the authors) in which we replaced the variable *whether or not the respondent has a child/children in several age categories* with separate variables on the *number of children* and the *age of the youngest child* yielded similar results as presented in Tables 3 to 5.

5. The same effects were significant when we entered the variables separately into the model.
